# The Efficacy of Denosumab in Patients With Rheumatoid Arthritis: A Systematic Review and Pooled Analysis of Randomized or Matched Data

**DOI:** 10.3389/fimmu.2021.799575

**Published:** 2022-01-05

**Authors:** Qiongwen Hu, Xue Zhong, Hua Tian, Pu Liao

**Affiliations:** ^1^Department of Medical Laboratory, Chongqing General Hospital, University of Chinese Academy of Sciences, Chongqing, China; ^2^Department of Nephrology, Chongqing General Hospital, University of Chinese Academy of Sciences, Chongqing, China

**Keywords:** denosumab, rheumatoid arthritis, bone mineral density, joint destruction, pooled analysis

## Abstract

**Objective:**

The purpose of this study was to evaluate the efficacy of denosumab treatment in patients with rheumatoid arthritis (RA).

**Methods:**

The Medline, Embase and Cochrane Library databases were searched for relevant clinical studies. Studies that assessed the efficacy of denosumab in patients with RA were identified. The primary endpoints were the percent changes in bone mineral density (BMD), and the changes in modified total Sharp score (mTSS), modified Sharp erosion score and joint space narrowing (JSN) score. Pooled analyses were calculated using random-effect models.

**Results:**

After searching the literature and performing further detailed assessments, 10 studies with a total of 1758 patients were included in the quantitative analysis. Pooled analyses showed that denosumab treatment significantly increased the percent changes in lumbar spine BMD [mean difference (MD): 5.12, confidence intervals (CI): 4.15 to 6.09], total hip BMD (MD: 2.72, 95% CI: 1.80 to 3.64) and femoral neck BMD (MD: 2.20, 95% CI: 0.94 to 3.46) compared with controls. Moreover, denosumab treatment significantly decreased the changes in mTSS (MD: -0.63, 95% CI: -0.86 to -0.41) and modified Sharp erosion score (MD: -0.62, 95% CI: -0.88 to -0.35). Subgroup analysis indicated that denosumab was superior to bisphosphonates for the improvement of BMD and the mitigation of joint destruction.

**Conclusion:**

Denosumab treatment was associated with increased BMD and alleviated progression of joint destruction in RA patients, even when compared with bisphosphonates.

## Introduction

Rheumatoid arthritis (RA) is a common autoimmune disease characterized by chronic inflammation of synovial joints, leading to the progression of joint destruction (1, 2). Multijoint destruction increases the risk of fractures in RA patients, and reduces the patient’s abilities of daily living and quality of life (3). Although there are various effective pharmacological therapies (such as conventional synthetic and biological disease-modifying anti-rheumatic drugs, DMARDs) that can abate joint inflammation and relieve joint destruction (2, 4), the joint-protective effect of these reagents is not complete. Additionally, infections and serious adverse events caused by the suppression of the immune system often occur (5, 6). Thus, the development of new therapies is essential for the treatment of RA.

Denosumab (AMG-162) is a fully human monoclonal antibody that specifically binds to human receptor activator of nuclear factor kappa B ligand (RANKL), resulting in decreased survival and activity of osteoclasts, thereby inhibiting bone resorption and bone loss (7, 8). It has been confirmed that denosumab is a highly effective and safe antiresorptive agent for the treatment of metastatic cancers and postmenopausal osteoporosis (9, 10). Several initial clinical trials have investigated the effect of denosumab treatment on patients with RA (11–14). The study by Kinoshita et al. showed that denosumab did not significantly suppress the progression of osteoporosis and the disease activity indices (12). Takeuchi et al. reported that denosumab significantly abated joint destruction and increased bone mineral density (BMD) compared with placebo (13). So et al. found that denosumab therapy was not associated with a significant improvement in erosion parameters at 12 months (14). Therefore, the findings of these studies regarding the efficacy of denosumab in RA remain to be further clarified.

In view of the discrepant findings of clinical studies, we conducted a systematic review and meta-analysis to evaluate the therapeutic effect of denosumab on BMD and joint destruction in patients with RA.

## Materials and Methods

### Search Methods and Sources

For identification of all published clinical studies that investigated the effects of denosumab on RA, we comprehensively searched the online published literature using the Medline, Embase and Cochrane Library databases (to October 7, 2021). The search strategy employed relevant keywords including the following: denosumab, AMG-162, RANKL inhibition and rheumatoid arthritis. The search scope was limited to English publications. To maximize the search for related studies, the reference lists of identified studies and systematic reviews were manually assessed. This study did not require ethics committee approval.

### Selection Criteria

(i) Randomized controlled trials (RCTs), matched prospective or matched retrospective studies that compared the efficacy of denosumab with controls in patients with RA; (ii) average age of patients ≥ 18 years; and (iii) reported data on the assessment of at least one of desired clinical endpoints: change in bone mineral density (BMD) and joint destruction scores. We tried to contact the corresponding authors to acquire further information when necessary data were not reported in the published articles. Two researchers (H.Q. and Z.X.) independently conducted the literature search, research eligibility assessment and data extraction.

### Quality Assessment

For the RCTs, we used the Cochrane Collaborative Risk of Bias tool (15) to assess the risk of bias in seven areas: allocation concealment; random sequence generation; blinding of research participants, outcome evaluators and medical service providers; incomplete outcome data; selective reports and other potential sources of bias. The quality of the included observational trials was assessed by the Newcastle–Ottawa Quality Assessment Scale (NOS) (16). Eight questions with nine possible points were included in the NOS scale. The data based on the comparability of the groups, the selection of populations, and the exposure/outcome of interest were judged using a star system. The RCTs and studies with NOS ≥ 7 were rated as being of good quality.

### Data Extraction

In the process of preparing this manuscript, the data extraction and presentation followed the recommendations of the Preferred Reporting Items for Systematic Reviews and Meta-Analyses (PRISMA, **Table S1**) (17) and the PICOS (population, intervention, comparison, outcome, study design, **Table S2**) framework (18). Two reviewers (H.Q. and Z.X.) used predefined standardized protocols and a data collection instrument to independently extract data from the included trials. Disagreements were resolved by consensus or the opinion of a third independent reviewer (L.P.).

### Outcome

The primary endpoints included percent changes from baseline in lumbar spine, total hip and femoral neck bone mineral density (BMD) and the changes from baseline in the modified total Sharp score (mTSS), the modified Sharp erosion score and the joint space narrowing (JSN) score. The mTSS has been used to evaluate the extent of bone erosions for 44 joints and JSN for 42 joints by scoring patient radiographs, with higher scores representing greater damage. This method was demonstrated to be sensitive enough to assess treatment effect over a short time for RA patients, including distal interphalangeal hand, wrist and feet joints (19). Secondary endpoints included American College of Rheumatology (ACR) 20/50/70 response (20), changes in the Health Assessment Questionnaire (HAQ; 0 = no difficulty; 3 = unable to do) (21), 28-joint count disease activity scores (DAS28) (22), serum C-telopeptide of type I collagen (CTX-I), serum N-propeptide of type I collagen (PINP), urine C-telopeptide of type II collagen (CTX-II)/creatinine and the incidence rates of serious adverse events.

### Statistical Analysis

We performed statistical analyses using RevMan software package 5.3 and STATA software 13.0. The analyses of continuous variables used weighted mean differences (MD) with 95% confidence intervals (CI), while the analyses of dichotomous data used relative risk (RR) with 95% CI. The Q-statistic was used to assess the existence of significant heterogeneity, and the I^2^ statistic was used to assess the degree of observed heterogeneity. A random effects model was used to calculate the pooled analyses. To detect any publication bias in the primary endpoints, we examined in detail the asymmetry of the funnel plots and further assessed them using the Begg adjusted rank correlation test and the Egger regression asymmetry test. To explore the influence of diverse covariates on the overall effect of denosumab on the primary endpoints of the percent changes in BMD and the changes in mTSS, we further performed sensitivity, subgroup and meta-regression analyses. A P value < 0.05 was considered statistically significant.

## Results

### Search Results and Study Qualities

**Figure 1:**
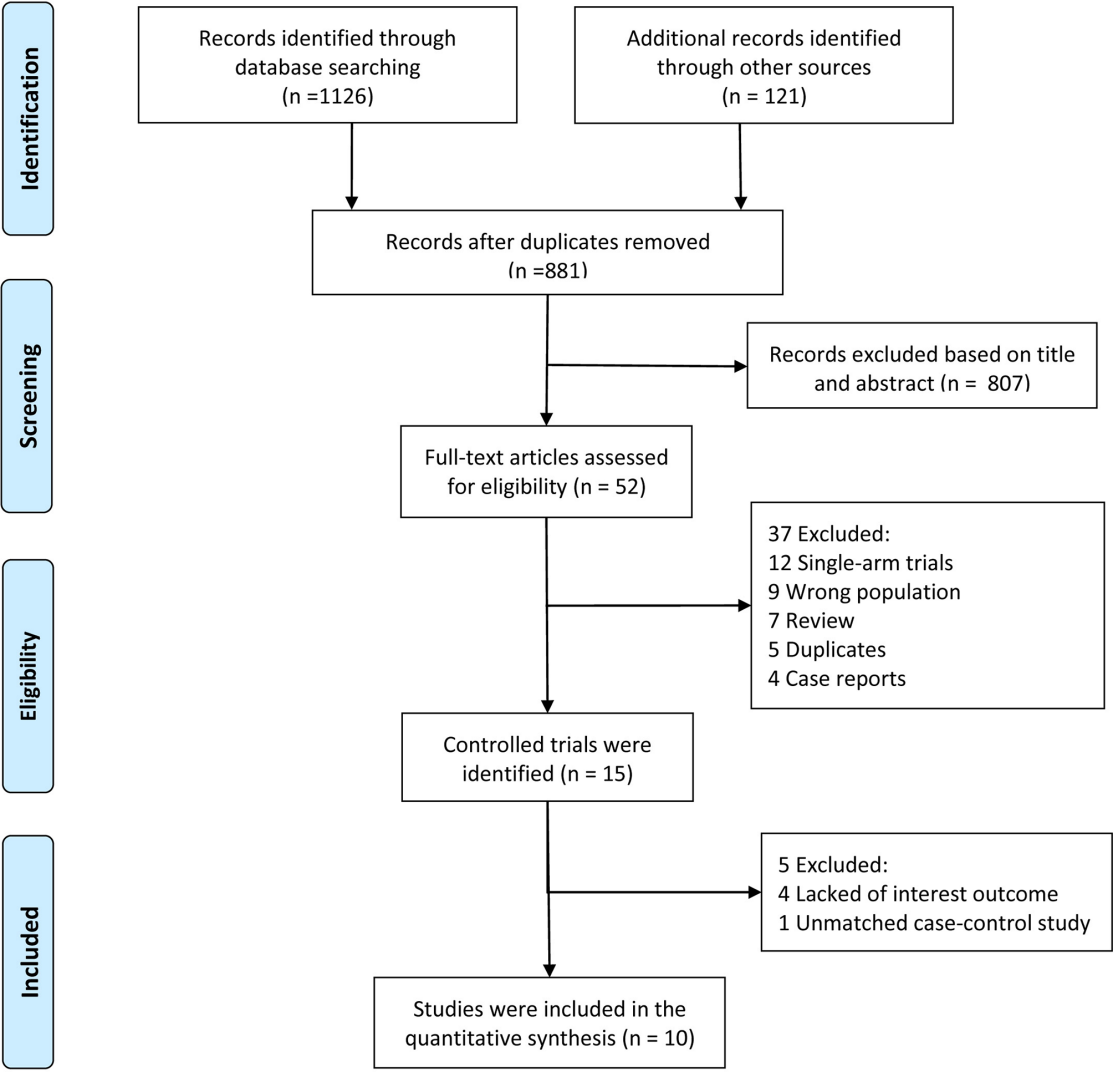
Flow chart of study selection.

Our systematic electronic literature search initially identified 881 studies. After reviewing the titles and abstract, 801 (91%) studies were excluded (**Figure 1**). Of the remaining 52 studies, 42 were excluded after a particular assessment of the full text for the following reasons: single-arm trials (n=12), incorrect populations (n=9), reviews (n=7), duplicates (n=5), case reports (n=4), lack of interesting outcomes (n=4) or unmatched case-control studies (n=1). After rigorous evaluation, 10 studies (5 RCTs and 5 matched studies) with a total of 1758 patients met our eligibility criteria for quantitative analysis (11–14, 23–28).

### Study Characteristics

The characteristics of the included studies were summarized in **Table 1**. Of these included patients, 1164 (66.2%) were women, with an average age of 59.6 years. The average disease duration was 6.2 years, and the average follow-up time was 13.0 months. In all studies, baseline characteristics were comparable between the denosumab group and the control group. Of these included studies, five studies were designed as RCTs (11, 13, 14, 23, 26), and 5 studies were designed as retrospective matched studies (12, 24, 25, 27, 28). Five studies compared denosumab with placebo (11, 13, 14, 23) or blank (24), whereas the remaining 5 studies compared denosumab with bisphosphonates (12, 25–28). Patients with RA were assigned to denosumab 60 mg every 6 months (Q6M) in 8 studies (11–14, 23–25, 28), 60 mg every 3 months (Q3M) in 2 studies (13, 23) and other doses in 3 studies (11, 23, 26). Detailed baseline data on the disease activity, severity and drug usage before the intervention were shown in **Table S3**. Except for one included study (27), all other studies were rated as being of good quality (**Table S4**).

**Table 1:** Baseline characteristics of the included studies.

Study (Ref.)	Year	Study Design	Simple Size, (N)	Women %	Age, Years	Duration, Years	Control	Denosumab, n, Dose	Follow-up, Months
Cohen et al. (11)	2008	RCT	218	73.1	57.4	11.0	Placebo	71, 60mg Q6M 72, 180mg Q6M	12
Takeuchi et al-a (23)	2016	RCT	340	78.2	54.5	2.3	Placebo	85, 60mg Q6M 82, 60mg Q3M 85, 60mg Q2M	12
Hasegawa et al. (24)	2017	Retrospective, matched study	80	93.8	72.2	13.1	Without denosumab	40, 60mg Q6M	12
Kinoshita et al. (12)	2017	Retrospective, matched study	98	94.9	69.2	12.8	Bisphosphonates	49,60mg Q6M	12
Nakamura1 et al. (25)	2017	Retrospective, matched study	52	100	70.2	15.4	Bisphosphonates	26, 60mg Q6M	24
Yue et al. (26)	2017	RCT	40	100	58.5	10.4	Bisphosphonate (alendronate)	20, 60mg Q1W	6
Ebina et al. (27)	2018	Retrospective, matched study	60	100	68.0	18.0	Bisphosphonates	30, NR	12
Takeuchi et al-b (13)	2019	RCT	654	74.8	57.4	2.2	Placebo	217, 60mg Q6M 219, 60mg Q3M	12
Mori1 et al. (28)	2021	Retrospective, matched study	106	100	69.4	10.5	Bisphosphonates	56, 60mg Q6M	12
So et al. (14)	2021	RCT	110	80.0	56.8	5.4	Placebo	55, 60mg Q6M	24

RCT, randomized controlled trial; Q6M, every 6 months; Q3M, every 3 months; Q2M, every 2 months; Q1W, every 1 week.

### Percent Changes in BMD

**Figure 2:**
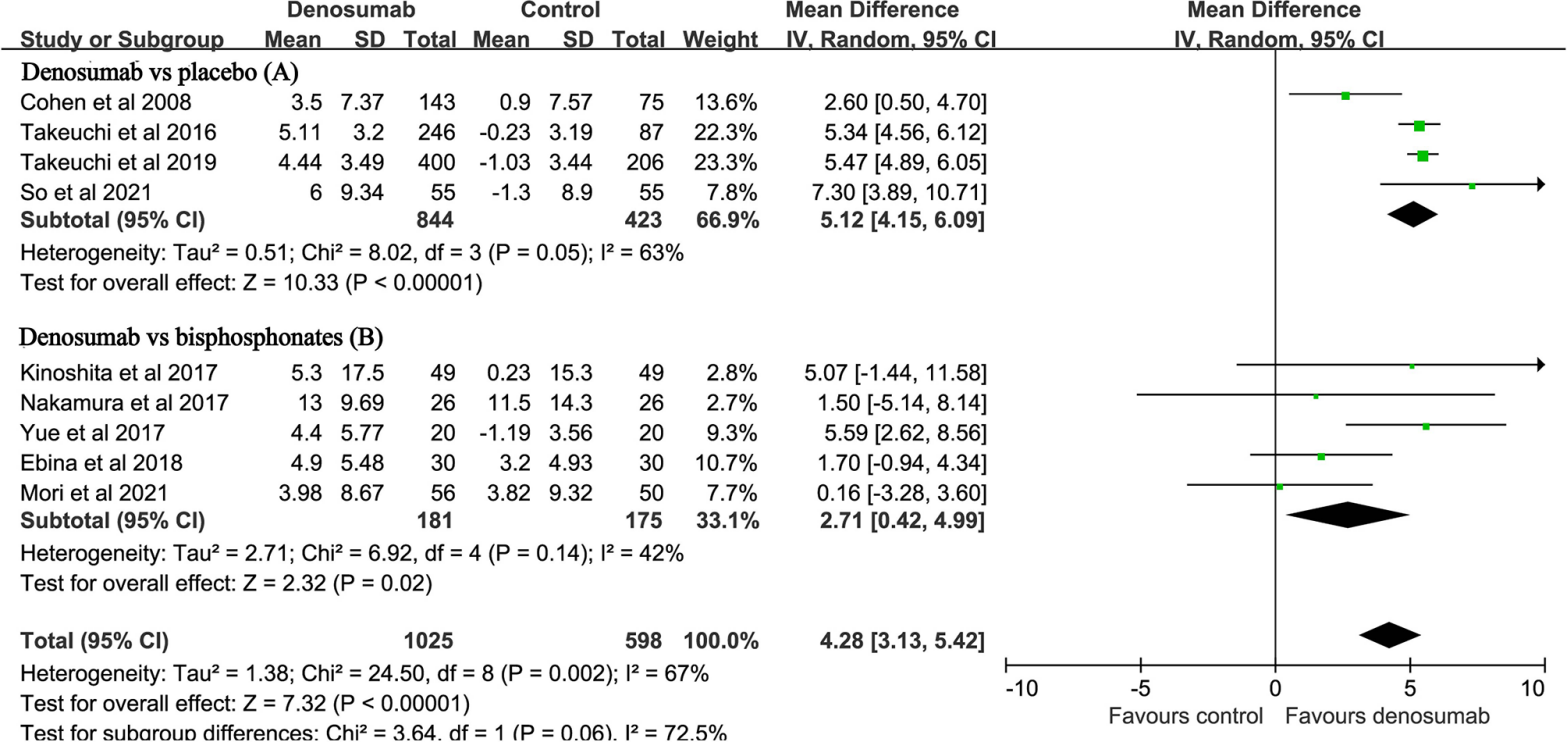
Forest plot for the effect of denosumab on the percent changes in lumbar spine BMD compared with placebo **(A)** and bisphosphonates **(B)**.

Nine studies with a total of 1623 patients reported the endpoint of percent changes in BMD at the lumbar spine (11–14, 23, 25–28). Pooled analyses showed that the percent changes in lumbar spine BMD were significantly higher in the denosumab group than in the control group (MD: 4.28, 95% CI: 3.13 to 5.42, P < 0.001, **Figure 2**). There was significant heterogeneity observed between these studies (P = 0.002, I^2^ = 67%). Compared with placebo, denosumab significantly increased the percent changes in lumbar spine BMD (MD: 5.12, 95% CI: 4.15 to 6.09, P<0.001; I^2^ = 63%, P = 0.05, **Figure 2A**). Compared with bisphosphonates, denosumab still increased the percent changes in lumbar spine BMD (MD: 2.71, 95% CI: 0.42 to 4.99, P = 0.02; I^2^ = 42%, P = 0.14, **Figure 2B**).

**Figure 3:**
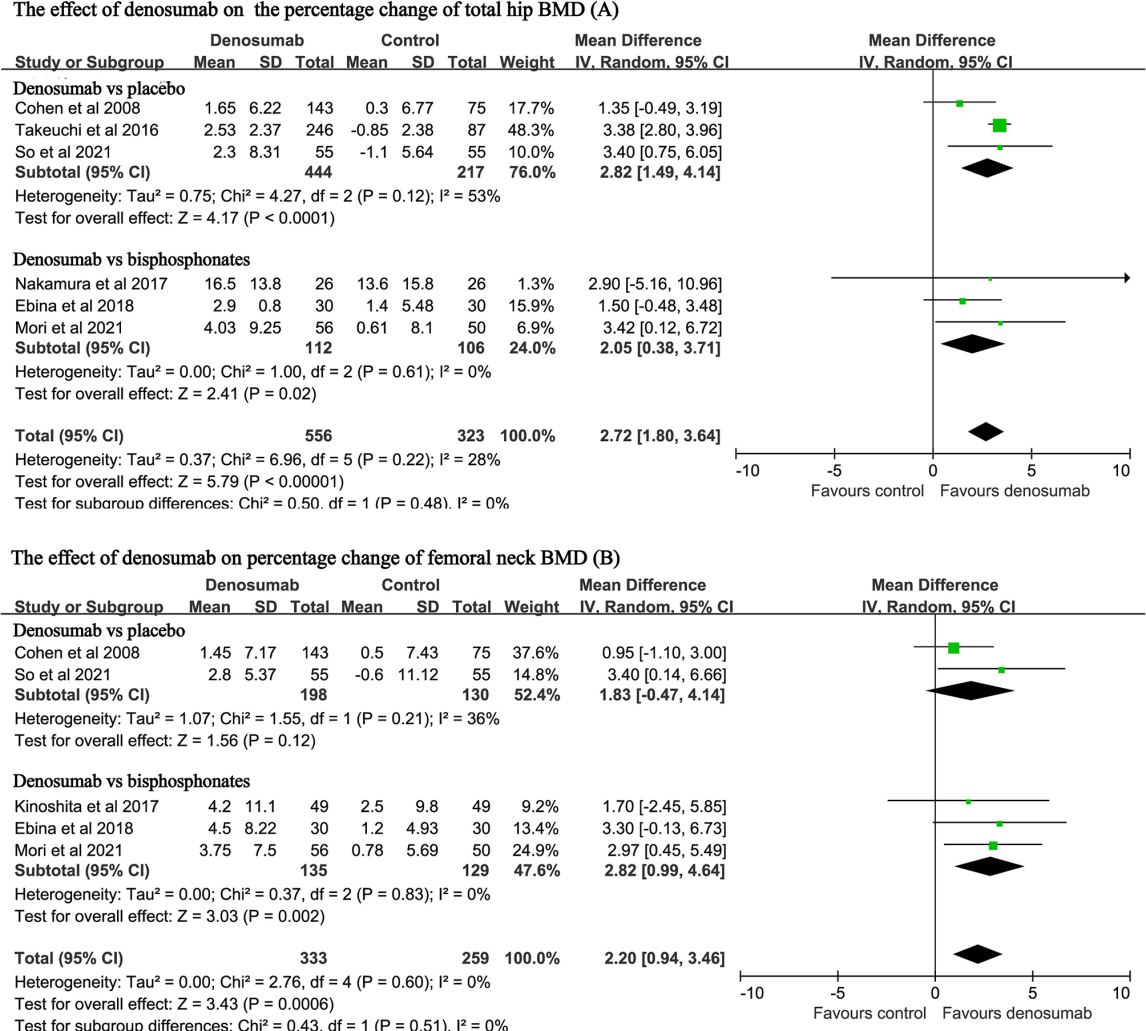
Forest plot for the effect of denosumab on the percent changes in total hip BMD **(A)** and femoral neck BMD **(B)**.

Six studies with a total of 879 patients reported the endpoint of percent changes in BMD at the total hip (11, 14, 23, 25, 27, 28). Pooled analyses showed that the percent change in total hip BMD was significantly higher in the denosumab group than in the control group (MD: 2.72, 95% CI: 1.80 to 3.64, P < 0.001, **Figure 3A**). There was mild heterogeneity observed between these studies (P = 0.22, I^2^ = 28%). Compared with placebo, denosumab significantly increased the percent changes in total hip BMD (MD: 2.82, 95% CI: 1.49 to 4.14, P < 0.001; I^2^ = 53%, P = 0.12). Compared with bisphosphonates, denosumab was still associated with increased percent changes in total hip BMD (MD: 2.05, 95% CI: 0.38 to 3.71, P = 0.02; I^2^ = 0, P = 0.61).

Five studies with a total of 592 patients reported the endpoint of percent changes in BMD at the femoral neck (11, 12, 14, 27, 28). Pooled analyses showed that the percent change in femoral neck BMD was significantly higher in the denosumab group than in the control group (MD: 2.20, 95%CI: 0.94 to 3.46, P < 0.001, **Figure 3B**). No heterogeneity was observed between these studies (P = 0.60, I^2^ = 0%). Compared with bisphosphonates, denosumab was still associated with increased percent changes in BMD at the femoral neck (MD: 2.82, 95% CI: 0.99 to 4.64, P = 0.002; I^2^ = 0, P = 0.83).

### Changes in the mTSS, the Erosion Score or the JSN Score

**Figure 4:**
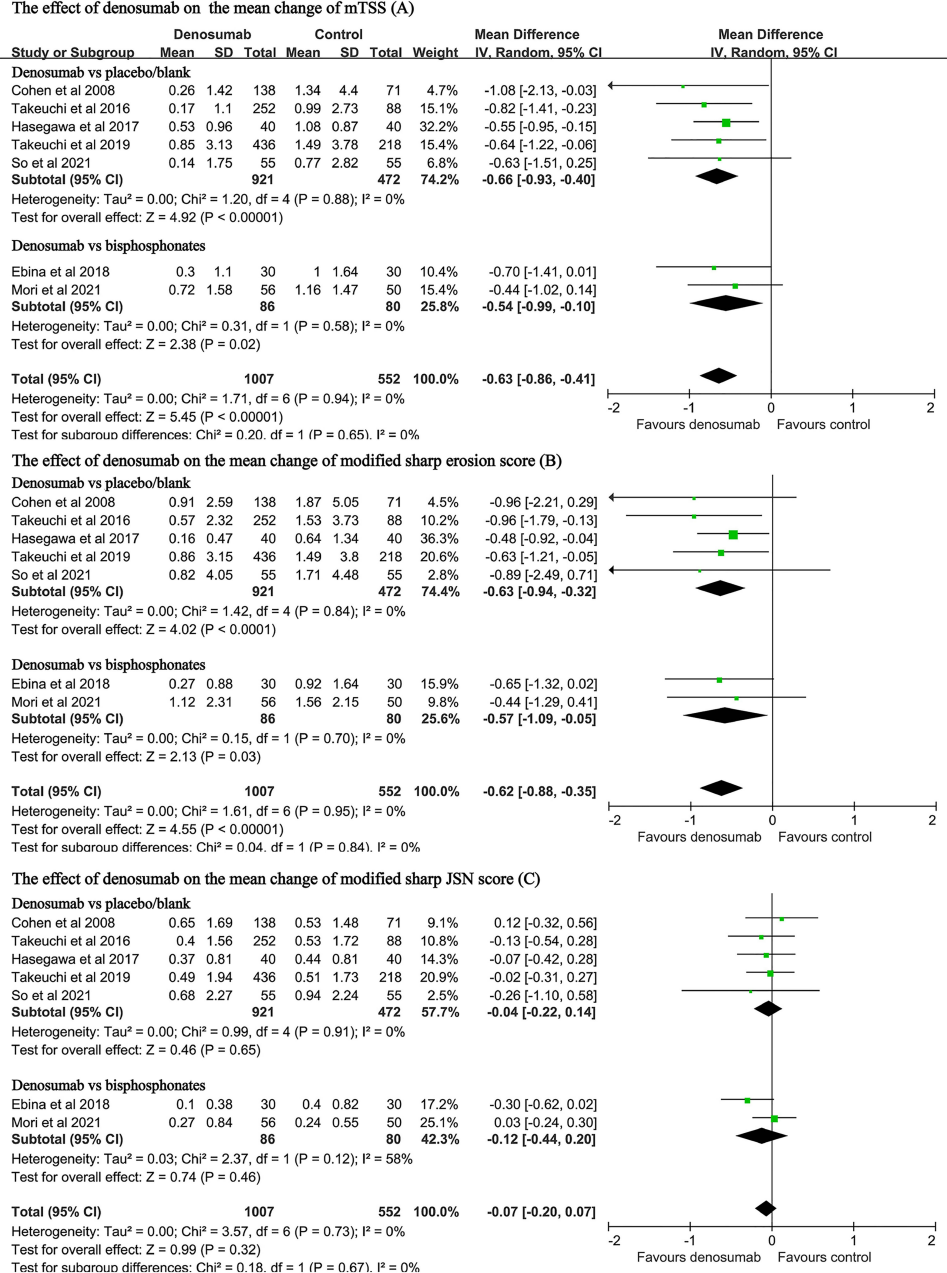
Forest plot for the effect of denosumab on the changes in mTSS **(A)**, modified sharp erosion score **(B)** and modified sharp JSN score **(C)**.

The data on these endpoints were available in seven studies with a total of 1559 patients (11, 13, 14, 23, 24, 27, 28). Pooled analyses found that, when compared with the control, denosumab treatment significantly decreased the changes in the mTSS (MD: -0.63, 95% CI: -0.86 to -0.41, P < 0.001; I^2^ = 0, P = 0.94, **Figure 4A**). Compared with placebo, denosumab significantly decreased the changes in the mTSS (MD: -0.66, 95%CI: -0.93 to -0.40, P < 0.001; I^2^ = 0, P = 0.88, **Figure 4A**). Compared with bisphosphonates, denosumab was still associated with significant reduction in the mTSS (MD: -0.54, 95%CI: -0.99 to -0.10, P = 0.02; I^2^ = 0, P = 0.58, **Figure 4A**).

Denosumab treatment also significantly decreased the changes in the modified Sharp erosion score (MD: -0.62, 95% CI: -0.88 to -0.35, P < 0.001; I^2^ = 0, P = 0.95, **Figure 4B**). Compared with bisphosphonates, denosumab was still associated with significant reduction in the modified Sharp erosion score (MD: -0.57, 95% CI: -1.09 to -0.05, P = 0.03; I^2^ = 0, P = 0.70, **Figure 4B**). However, denosumab treatment was not associated with significant changes regarding the JSN score in either the placebo-controlled subgroup (MD: -0.04 95% CI: -0.22 to 0.14, P=0.65; I^2^ = 0, P = 0.91, **Figure 4C**) or the bisphosphonate-controlled subgroup (MD: -0.12, 95% CI: -0.44 to 0.20, P = 0.46; I^2^ = 58%, P = 0.12, **Figure 4C**).

### Secondary Endpoints

Compared with the control, denosumab treatment did not significantly change the HAQ scores (MD: -0.08, 95% CI: -0.17 to 0.01, P = 0.09, **Figure S1A**), DAS28 scores (MD: -0.01, 95% CI: -0.05 to 0.02, P = 0.39; **Figure S1B**), ACR20 response (OR: 1.13, 95% CI: 0.87 to 1.47, P = 0.37, **Figure S2A**), ACR50 response (OR: 1.07, 95% CI: 0.67 to 1.73, P = 0.77, **Figure S2B**) and ACR70 response (OR: 1.02, 95%CI: 0.57 to 1.84, P = 0.94, **Figure S2C**). Pooled analyses showed that denosumab treatment substantially suppressed the markers of bone turnover serum CTX-I (MD: -50.69, 95% CI: -64.18 to -37.20, P < 0.001, **Figure S3A**), urine CTX-II/creatinine (MD: -38.59, 95% CI: -57.30 to -19.88, P < 0.001, **Figure S3B**) and PINP (MD: -39.77, 95% CI: -56.10 to -23.43, P < 0.001, **Figure S3C**). Of these included studies, six studies provided the data on adverse events (11, 13, 14, 23, 25, 28), and detailed information were shown in **Table S5**. Pooled analysis indicated that the incidence rates of serious adverse events between the denosumab and control groups were comparable (OR: 0.99, 95% CI: 0.63 to 1.55, P = 0.96; **Figure S4**).

### Sensitivity, Subgroup and Meta-Regression Analyses

**Figure 5:**
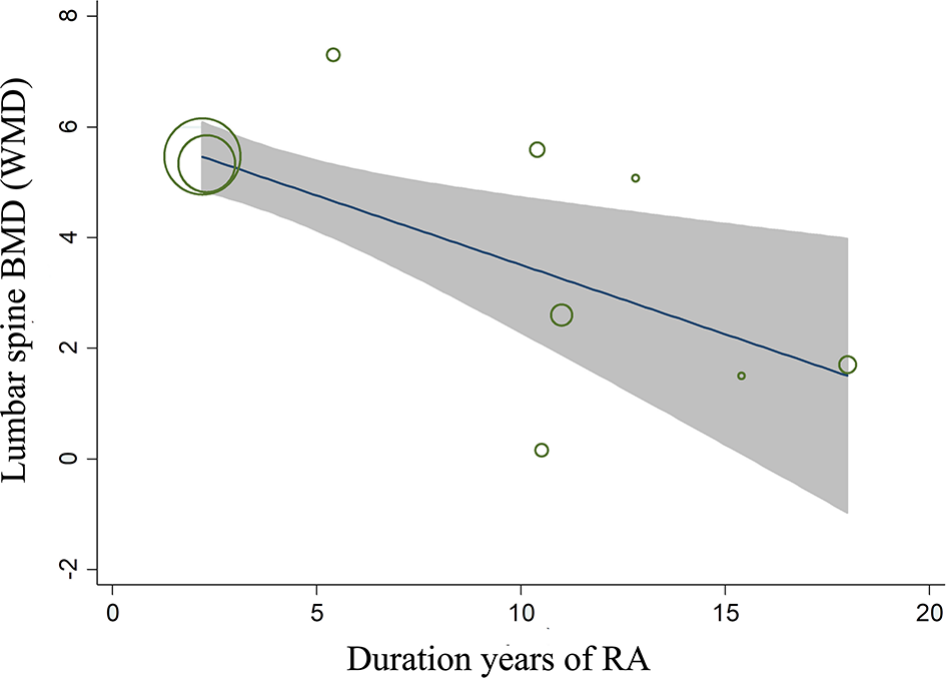
Meta-regression analysis between the duration of RA and the effect of denosumab on the percent changes in lumbar spine BMD.

Sensitivity analysis (using the single-study-removed method) indicated good stability in the primary endpoints of percent changes from baseline in lumbar spine BMD and changes from baseline in the mTSS (**Figure S5**). In the subgroup analysis, denosumab treatment was still associated with increased percent changes in lumbar spine BMD and decreased changes in the mTSS, except for the case–control subgroup in lumbar spine BMD (**Table 2**). For the endpoint of percent changes in lumbar spine BMD, meta-regression indicated that the duration of RA was the major source of heterogeneity of denosumab treatment (P < 0.01, **Table S6**). This result indicated that the effect of denosumab on percent changes in lumbar spine BMD may be negatively correlated with the duration of RA (**Figure 5**). The sex, glucocorticoids use, baseline lumbar spine BMD, positive rheumatoid factor and denosumab dose were not significantly correlated with the major source of heterogeneity (**Table S6**).

**Table 2:** Outcomes of subgroup analysis.

Endpoint	Subgroup	No. of Studies (N)	MD (95% CI)	*P*-value	*I^2^* (%)
LS-BMD	RCT*	5 (1307)	5.18 (4.33 – 6.03)	<0.001	50
	Case-control^#^	4 (316)	1.50 (-0.41 – 3.41)	0.12	0
	Duration < 10 y	3 (1049)	5.46 (5.00 – 5.92)	<0.001	0
	Duration ≥ 10 y	6 (574)	2.67 (1.07 – 4.27)	0.001	28
	60mg, Q6M	7 (1087)	3.97 (2.67 – 5.27)	<0.001	67
	60mg, Q3M	2 (575)	5.70 (5.11 – 6.28)	<0.001	9
	Other dose	3 (357)	5.03 (2.89 – 7.17)	<0.001	75
mTSS	RCT*	4 (1313)	-0.75 (-1.10 – -0.40)	<0.001	0
	Case-control^#^	3 (246)	-0.55 (-0.85 – -0.25)	<0.001	0
	Duration < 10 y	3 (1104)	-0.71 (-1.08 – -0.34)	<0.001	0
	Duration ≥ 10 y	4 (455)	-0.59 (-0.88 – -0.30)	<0.001	0
	60mg, Q6M	6 (1044)	-0.58 (-0.83 – -0.33)	<0.001	0
	60mg, Q3M	2 (607)	-0.81 (-1.22 – -0.40)	<0.001	0
	Other dose	2 (313)	-0.97 (-1.53 – -0.42)	<0.001	0

LS-BMD, lumbar spine-bone mineral density; mTSS, modified total Sharp score; MD, mean differences; Q3M, 60 mg every 3 months; Q6M, 60 mg every 6 months; RCT, randomized controlled trials; *age < 65 years; ^＃^age ≥ 65 years.

### Publication Bias

For the primary endpoints of percent changes from the baseline in BMD and the changes in the mTSS, funnel plots of all included studies displayed symmetry, showing a low risk of publication bias (**Figure S6**). For Begg’s and Egger’s tests, potentially significant publication bias did not exist for each primary endpoint (*P_begg_*=0.13 and *P_egger_*=0.14 for percent changes in BMD; *P_begg_*=0.25 and *P_egger_*=0.12 for changes in the mTSS).

## Discussion

To our knowledge, this is the first systematic review and pooled-analysis to analyze the efficacy of denosumab in patients with RA. On the grounds of the available published evidence, we found that denosumab treatment significantly increased lumbar spine, total hip and femoral neck BMD in RA patients. Denosumab treatment significantly decreased the changes in the mTSS and the modified Sharp erosion score. Although denosumab treatment did not significantly change the HAQ scores, DAS28 scores and ACR20/50/70 responses, it suppressed serum CTX-I, PINP and urine CTX-II/creatinine level. Additionally, denosumab treatment was not associated with an increased risk of serious adverse events.

Patients with RA have significantly increased risks of bone loss and fractures (29). In this study, we found that denosumab increased lumbar spine BMD by 5.12% (4.15 to 6.09) and total hip BMD by 2.82% (1.49 to 4.14) in RA patients compared with placebo. Takeuchi et al. reported that regardless of whether the patient was taking glucocorticoids or suffering from osteoporosis, an increase (5.47%) in lumbar spine BMD was observed during denosumab treatment in RA (13, 23), which was consistent with our findings. Patients with rheumatic diseases were at high risk of systemic bone loss and osteoporotic fractures and were suggested to be treated with bisphosphonates (30, 31). Our data indicated that denosumab appeared to have a better effect on increasing lumbar spine, total hip and femoral neck BMD than bisphosphonates, for a difference of 2.71%, 2.05% and 2.82% respectively. These discrepant effects between denosumab and bisphosphonates may be explained by their distributions and mechanisms of action. Although bisphosphonates have an antiresorption effect by acting on osteoclasts, denosumab directly targets the production of osteoclasts through its specific effect on the RANKL pathway (32).

Subgroup analysis indicated that denosumab treatment appeared to be more effective to increase lumbar spine BMD in RCTs than in observational studies. The probable reason was that most of these RCTs had a large sample size and were compared with placebo. Although meta-regression analysis indicated that the effect of denosumab on the percent change in lumbar spine BMD was negatively correlated with the duration of RA, we could not rule out the interaction between diverse variables, especially age and RA severity. Whether denosumab should be administered in the early stage of RA still needs further study. A statistically significant dose-response curve had not been observed in meta-regression analysis. The possible reason was that most of the RA patients included in our data administered denosumab with Q6M, while fewer patients administered denosumab with other dosage.

RA is a systemic autoimmune inflammatory disease, which leads to osteoporosis and joint destruction by activating osteoclasts (2, 33). The joint damage of RA is irreversible and is closely related to clinical outcomes (3, 34). Consequently, the prevention of joint destruction is vital to alleviate the progression of RA. Our data showed that denosumab treatment was associated with significantly smaller changes in the mTSS (-0.66, -0.93 to -0.40) and the modified Sharp erosion score (-0.63, -0.94 to -0.32) compared to placebo, which were consistent with the results (-0.70 to -0.54) of several previous single-arm studies (35, 36). Furthermore, when compared with bisphosphonates, the therapeutic effect of denosumab on joint destruction was not significantly weakened. Subgroup analysis showed that denosumab treatment effectively reduced the mTSS in different study designs (RCTs or observational studies), dosage (Q6M, Q3M or other) and disease duration. The study by So et al. found that significant radiological changes in the mTSS and the modified Sharp erosion score could not be detected in the RA patients treated with denosumab (14), which may be caused by the small sample size. Although denosumab suppressed joint margin erosion, it did not block the changes in JSN. This may be related to the mechanism of denosumab, suggesting that denosumab may have no inhibitory effect on cartilage destruction (13, 37).

For the changes in DAS28 scores, HAQ scores and any component of ACR response, no clinically meaningful differences were observed between the denosumab and control groups. These findings showed that denosumab might have no effect on the activity of RA disease, which was consistent with previous reports (28, 38). Additionally, the mean 13-month follow-up may be too short to achieve low disease activity or remission in patients with RA. More long-term studies need to further clarify the efficacy of denosumab on functional disability.

In the present study, we found that bone turnover markers (CTX-I and PINP) and cartilage markers (urine CTX-II/Cre) were suppressed by denosumab treatment. The suppression of urine CTX-II/Cre suggested the possibility that the use of denosumab to prevent bone destruction might lead to secondary inhibition of cartilage destruction. However, Takeuchi et al. and So et al. found that denosumab did not affect the cartilage turnover marker serum cartilage oligomeric protein (COMP) (13, 14), indicating that denosumab might have no anti-inflammatory effect on RA patients. Our data found that denosumab was not associated with increased risk of serious adverse events. However, recently, several studies reported that the rapid bone loss and the rebound fractures may occur when treatment is stopped (39, 40), which needs to be considered when choosing this agent.

Our study had several limitations: First, the mean 13-month follow-up might be too short to fully clarify the effect of denosumab on the healing of erosions and its effect on functional disability. Long-term follow up studies (for example, 5 years) are still needed. Second, five of these included studies were retrospective, which led to possible biases in our results. Although we tried to overcome this limitation by performing multiple-sensitivity, subgroup and meta-regression analyses, potential bias could still not be ruled out. Third, only five studies with a total of 356 patients compared the efficacy of denosumab with bisphosphonates. Whether denosumab treatment is superior to bisphosphonates in patients with RA requires confirmation in a larger RCT. Finally, the patients included in this study were mainly from Japan, and potential racial bias cannot be ruled out.

In conclusion, data from our meta-analysis indicated that denosumab treatment was associated with increased lumbar spine and total hip BMD in patients with RA. Denosumab treatment decreased the changes in the mTSS and the modified Sharp erosion score. Additionally, denosumab may be superior to bisphosphonates for the prevention of osteoporosis and bone erosion.

## Data Availability Statement

The original contributions presented in the study are included in the article/**Supplementary Material**. Further inquiries can be directed to the corresponding author.

## Author Contributions

All authors participated in the drafting or critical revision of this article and contributed important intellectual content. All authors read and approved the final version of the manuscript. QH is responsible for the completeness of the data and the accuracy of data analysis. QH, XZ and PL commented on the study concept and design. QH and XZ carried out the acquisition of data. QH, HT and PL performed the analysis and interpretation of data. All authors contributed to the article and approved the submitted version.

## Conflict of Interest

The authors declare that the research was conducted in the absence of any commercial or financial relationships that could be construed as a potential conflict of interest.

## Publisher’s Note

All claims expressed in this article are solely those of the authors and do not necessarily represent those of their affiliated organizations, or those of the publisher, the editors and the reviewers. Any product that may be evaluated in this article, or claim that may be made by its manufacturer, is not guaranteed or endorsed by the publisher.
