# Supplementary material for: The Efficacy of Denosumab in Patients With Rheumatoid Arthritis: A Systematic Review and Pooled Analysis of Randomized or Matched Data
Source: Front Immunol. 2022 Jan 5;12:799575. doi: 10.3389/fimmu.2021.799575 (PMC8766643; doi:10.3389/fimmu.2021.799575)
Supplement: Supplementary file 1 [file DataSheet_1.docx]

**Supplementary Materials List**

Table S1. PRISMA Checklist.

Table S2. PICOS checklist.

Table S3. Baseline demographics in the included studies.

Table S4. Assessment of study quality.

Table S5. Summary of adverse events.

Table S6. Meta-regression analysis for the influence of diverse variables in the effect of denosumab on lumbar spine BMD.

Figure S1. Forest plot for the effect of denosumab on the changes in HAQ scores (A) and DAS28 scores (B).

Figure S2. Forest plot for the effect of denosumab on ACR20 response (A), ACR50 response (B) and ACR70 response (C).

Figure S3. Forest plot for the effect of denosumab on serum CTX-I (A), urine CTX-II/creatinine (B) and serum PINP (C).

Figure S4. Forest plot for incidence rates of serious adverse events.

Figure S5. Sensitivity analysis for the effect of denosumab on the changes in lumbar spine BMD (A) and mTSS (B).

Figure S6. Funnel plots of all included studies.

Table S1. PRISMA Checklist.

| **Section/topic** | **#** | **Checklist item** | **Reported on page #** |
| --- | --- | --- | --- |
| **TITLE** | | |  |
| Title | 1 | Identify the report as a systematic review, meta-analysis, or both. | Title (page 1) |
| **ABSTRACT** | | |  |
| Structured summary | 2 | Provide a structured summary including, as applicable: background; objectives; data sources; study eligibility criteria, participants, and interventions; study appraisal and synthesis methods; results; limitations; conclusions and implications of key findings; systematic review registration number. | Abstract  (page 2-3) |
| **INTRODUCTION** | | |  |
| Rationale | 3 | Describe the rationale for the review in the context of what is already known. | Introduction  (page 4) |
| Objectives | 4 | Provide an explicit statement of questions being addressed with reference to participants, interventions, comparisons, outcomes, and study design (PICOS). | Introduction  (page 5) |
| **METHODS** | | |  |
| Protocol and registration | 5 | Indicate if a review protocol exists, if and where it can be accessed (e.g., Web address), and, if available, provide registration information including registration number. | None |
| Eligibility criteria | 6 | Specify study characteristics (e.g., PICOS, length of follow-up) and report characteristics (e.g., years considered, language, publication status) used as criteria for eligibility, giving rationale. | Methods  (page 5-6) |
| Information sources | 7 | Describe all information sources (e.g., databases with dates of coverage, contact with study authors to identify additional studies) in the search and date last searched. | Methods  (page 5) |
| Search | 8 | Present full electronic search strategy for at least one database, including any limits used, such that it could be repeated. | Methods  (page 5) |
| Study selection | 9 | State the process for selecting studies (i.e., screening, eligibility, included in systematic review, and, if applicable, included in the meta-analysis). | Methods  (page 5) |
| Data collection process | 10 | Describe method of data extraction from reports (e.g., piloted forms, independently, in duplicate) and any processes for obtaining and confirming data from investigators. | Methods  (page 6) |
| Data items | 11 | List and define all variables for which data were sought (e.g., PICOS, funding sources) and any assumptions and simplifications made. | Methods  (page 6-7) |
| Risk of bias in individual studies | 12 | Describe methods used for assessing risk of bias of individual studies (including specification of whether this was done at the study or outcome level), and how this information is to be used in any data synthesis. | Methods  (page 6) |
| Summary measures | 13 | State the principal summary measures (e.g., risk ratio, difference in means). | Methods  (page 7-8) |
| Synthesis of results | 14 | Describe the methods of handling data and combining results of studies, if done, including measures of consistency (e.g., I^2^) for each meta-analysis. | Methods  (page 7-8) |
| Section/topic | # | Checklist item | Reported on page # |
| Risk of bias across studies | 15 | Specify any assessment of risk of bias that may affect the cumulative evidence (e.g., publication bias, selective reporting within studies). | Methods  (page 8) |
| Additional analyses | 16 | Describe methods of additional analyses (e.g., sensitivity or subgroup analyses, meta-regression), if done, indicating which were pre-specified. | Methods  (page 8) |
| **RESULTS** | | |  |
| Study selection | 17 | Give numbers of studies screened, assessed for eligibility, and included in the review, with reasons for exclusions at each stage, ideally with a flow diagram. | Results  (page 8) |
| Study characteristics | 18 | For each study, present characteristics for which data were extracted (e.g., study size, PICOS, follow-up period) and provide the citations. | Results  (page 8-9) |
| Risk of bias within studies | 19 | Present data on risk of bias of each study and, if available, any outcome level assessment (see item 12). | Results  (page 9) |
| Results of individual studies | 20 | For all outcomes considered (benefits or harms), present, for each study: (a) simple summary data for each intervention group (b) effect estimates and confidence intervals, ideally with a forest plot. | Results  (page 7-8) |
| Synthesis of results | 21 | Present results of each meta-analysis done, including confidence intervals and measures of consistency. | Results  (page 9-12) |
| Risk of bias across studies | 22 | Present results of any assessment of risk of bias across studies (see Item 15). | Results  (page 12-13) |
| Additional analysis | 23 | Give results of additional analyses, if done (e.g., sensitivity or subgroup analyses, meta-regression [see Item 16]). | Results  (page 12) |
| **DISCUSSION** | | |  |
| Summary of evidence | 24 | Summarize the main findings including the strength of evidence for each main outcome; consider their relevance to key groups (e.g., healthcare providers, users, and policy makers). | Discussion  (page 13-16) |
| Limitations | 25 | Discuss limitations at study and outcome level (e.g., risk of bias), and at review-level (e.g., incomplete retrieval of identified research, reporting bias). | Discussion  (page 16-17) |
| Conclusions | 26 | Provide a general interpretation of the results in the context of other evidence, and implications for future research. | Discussion  (page 17) |
| **FUNDING** | | |  |
| Funding | 27 | Describe sources of funding for the systematic review and other support (e.g., supply of data); role of funders for the systematic review. | None |

Table S2. PICOS checklist (Population, Intervention, Control, Outcome, Study Design (PICOS) inclusion criteria)

| **Population** | Patients diagnosed with rheumatoid arthritis based on the American College of Rheumatology criteria |
| --- | --- |
| **Intervention** | Denosumab 180mg Q6M, 60mg Q6M, 60mg Q3M, 60mg Q2M, 60mg Q1W |
| **Control** | Placebo, blank or bisphosphonates |
| **Outcomes** | Percent changes in lumbar spine, total hip and femoral neck BMD and the changes in the modified total Sharp score, the modified sharp erosion score and the joint space narrowing score |
| **Study design** | Randomsied controlled trials, matched prospective or matched retrospective studies |

Abbreviations: Q6M: every 6 months, Q3M: every 3 months, Q2M: every 2 months, Q1W: every 1 week.

Table S3. Baseline demographics in the included studies.

| **Study  (Ref. #)** | **RF (+),  (%)** | **MTX  use, %** | **ACPA (+), (%)** | **GLU use , (%)** | **NSAIDs  use, %** | **DMARDs use, %** | **HAQ-DI** | **LS-BMD , g/cm2** | **TH-BMD , g/cm2** | **FN-BMD , g/cm2** | **mTSS score** | **MSE score** | **MS-JSN score** | **DAS28 -CRP** |
| --- | --- | --- | --- | --- | --- | --- | --- | --- | --- | --- | --- | --- | --- | --- |
| Cohen et al [11] | 78.0/77.2 | NR | NR | 35.9/37.6 | NR | 2.6/2.7 | NR | NR | NR | NR | 29.9/45.8 | 16.6/26.1 | 13.3/19.6 | NR |
| Takeuchi  et al-a [23] | 68.2/68.2 | NR | 75.0/79.0 | 42.0/43.7 | 72.7/75.0 | 25.0/21.8 | 0.47/0.35 | 1.04/1.07 | 0.83/0.86 | NR | 13.6/11.4 | 6.6/6.6 | 6.94/4.82 | 3.95/3.72 |
| Hasegawa  et al [24] | 77.5/70.0 | 55/47.5 | NR | 37.5/42.5 | NR | NR | 0.70/1.24 | NR | NR | NR | 12.3/24.3 | 43.8/48 | 33.3/25.0 | 3.1/2.94 |
| Kinoshita  et al [12] | NR | NR | NR | NR | NR | NR | NR | NR | NR | NR | NR | NR | NR | 2.85/3.07 |
| Nakamura1  et al [25] | NR | NR | NR | NR | NR | 34.6/30.8 | 0.6/0.7 | 0.70/0.70 | 0.50/0.50 | NR | NR | NR | NR | 3.5/3.8 |
| Yue et al [26] | 75/85 | 80/85 | 60.0/50.0 | 15/25 | 50/50 | 5/10 | NR | 0.45/0.45 | NR | NR | NR | NR | NR | 3.3/3.1 |
| Ebina et al [27] | 85.0/90.0 | 78.4/86.7 | 85.0/86.7 | 63.4/56.7 | NR | NR | NR | 2.0/2.0 | 2.0/2.35 | 2.4/2.6 | 91.6/77.8 | 35.6/32.7 | 56/45.1 | 2.4/2.2 |
| Takeuchi  et al-b [13] | 62.8/61.5 | 87.2/83.7 | 66.5/70.4 | 31.7/32.3 | 66.5/69.5 | NR | 0.31/0.39 | 1.11/1.02 | NR | NR | 13.1/15.5 | 6.55/7.34 | 6.59/8.20 | 3.43/3.60 |
| Mori1  et al [28] | NR | 76/46.4 | 100/100 | 44/32.1 | NR | NR | 0.52/0.64 | 0.89/0.86 | 0.69/0.67 | 0.58/0.56 | 60.1/52.5 | 36.1/29.6 | 24.1/23.1 | 3.1/2.83 |
| So et al [14] | 72/69 | 76/87 | 78/80 | 11/11 | 53/64 | 11/16 | 0.13/0.25 | 0.91/0.93 | 0.84/0.85 | 0.68/0.70 | 9/8 | 1/2 | 4/6 | 2.43/2.60 |
| “/”, denosumab vs control; ACPA, anticyclic citrullinated peptide antibody; BMD, bone mineral density; CRP, C reactive protein; DAS, disease activity score; DMARDs, disease-modifying antirheumatic drugs; FN, femoral neck; GLU, glucocorticoid; HAQ-DI, Health Assessment Questionnaire-Disability Index; JSN, joint space narrowing; LS, lumbar spine; MS, Modified Sharp; MTX, methotrexate; mTSS, modified total Sharp score; MSE, Modified Sharp erosion score; NR, not record; NSAID, non-steroidal anti-inflammatory drug; RF, rheumatoid factor; TH, total hip. | | | | | | | | | | | | | | |

Table S4. Assessment of study quality.

| **RCTs** | **Quality indicators from Cochrane Collaboration’s risk of bias tool** | | | | | | | | | | | | | | |
| --- | --- | --- | --- | --- | --- | --- | --- | --- | --- | --- | --- | --- | --- | --- | --- |
| **Ref.** | **Random sequence generation** | | **Allocation concealment** | | | **Blinding of participants and personnel** | | **Blinding of Outcome assessment** | | **No incomplete data** | | **No selective reporting** | | | **No other**  **bias** |
| Cohen et al [11] | Unclear | | Yes | | | Yes | | Yes | | Yes | | Yes | | | Unclear |
| Takeuchi et al-a [23] | Yes | | Yes | | | Yes | | Yes | | Yes | | Yes | | | Unclear |
| Yue et al [26] | Yes | | Unclear | | | Unclear | | Yes | | Yes | | Yes | | | Unclear |
| Takeuchi et al-b [13] | Yes | | Yes | | | Yes | | Yes | | Yes | | Yes | | | No |
| So et a [14] | Yes | | Yes | | | Yes | | Yes | | Yes | | Yes | | | Yes |
| **Matched studies** | **Quality indicators from NOS** | | | | | | | | | | | | |  | |
| **Ref.** | **Selection Comparability Exposure/outcome Score** | | | | | | | | | | | | | | |
| Hasegawa et al [24] | Yes | No | | No | Yes | | Yes | Yes | Yes | | Yes | | Yes | 7 | |
| Kinoshita et al [12] | Yes | Yes | | No | Yes | | No | Yes | Yes | | Yes | | Yes | 7 | |
| Nakamura1 et al [25] | Yes | No | | No | Yes | | Yes | Yes | Yes | | Yes | | Yes | 7 | |
| Ebina et al [27] | Yes | No | | No | Yes | | Yes | No | Yes | | Yes | | Yes | 6 | |
| Mori1 et al [28] | Yes | Yes | | No | No | | Yes | Yes | Yes | | Yes | | Yes | 7 | |
| NOS: Newcastle-Ottawa quality assessment Scale. For case-control studies: (1) represents cases with independent validation; (2) cases are consecutive or representative; (3) controls are community; (4) controls have no history of prior use of denosumab; (5) study controls are comparable for age and sex; (6) study controls for any additional factor(s); (7) cases and controls have the same method of ascertainment; (8) was follow-up long enough for outcomes to occur; and (9) cases and controls have complete follow-up. RCT: Randomized controlled trial. | | | | | | | | | | | | | | | |

Table S5. Summary of adverse events.

| **Study** | **Cohen et al 2008 [11]** | | **Takeuchi et al-a 2016 [23]** | | **Nakamura1 et al [25]** | | **Takeuchi et al-b 2019 [13]** | | **Mori1 et al 2021 [28]** | | **So et al 2021 [14]** | |
| --- | --- | --- | --- | --- | --- | --- | --- | --- | --- | --- | --- | --- |
| **Groups**  **Adverse events** | Placebo  N=75 | Dmab  N=143 | Placebo  N=88 | Dmab  N=258 | Placebo  N=26 | Dmab  N=26 | Placebo  N=226 | Dmab  N=453 | BP  N=50 | Dmab  N=56 | Placebo  N=55 | Dmab  N=55 |
| **Serious adverse event** | 7 (9.3) | 9 (6) | 9 (10) | 18 (7) | 0 (0) | 0 (0) | 13 (5.8) | 38 (8.6) | 2 (4) | 1 (1.8) | 0 (0) | 1 (1.8) |
| **Discontinuation due to adverse event** | 1 (1) | 1 (1) | 2 (4.5) | 8 (3.1) | NR | NR | 4 (1.8) | 11 (2.4) | NR | NR | NR | NR |
| **Treatment-related adverse event** | 7 (9) | 18 (13) | 16 (18) | 46 (18) | NR | NR | NR | NR | NR | NR | NR | NR |
| **Treatment-related serious adverse event** | 0 (0) | 0 (0) | 2 (2) | 3 (1) | 0 (0) | 0 (0) | 3 (1.3) | 9 (2.0) | NR | NR | NR | NR |
| **Fracture** | NR | NR | 0 (0) | 0 (0) | 0 (0) | 0 (0) | NR | NR | 2 (4) | 1 (1.8) | NR | NR |
| **Death** | 0 (0) | 0 (0) | 0 (0) | 0 (0) | 0 (0) | 0 (0) | 0 (0) | 1 (0.2) | 0 (0) | 0 (0) | 0 (0) | 0 (0) |
| Dmab: denosumab, NR: not record. | | | | | | | | | | | | |

Table S6. Meta-regression analysis for the influence of diverse variables in the effect of denosumab on lumbar spine BMD.

| **Variables** | **Study, (n)** | **Adjusted R-Square, %** | **95%CI** | **P-value** |
| --- | --- | --- | --- | --- |
| Duration of RA, years | 9 | 100.0 | (-0.417 to -0.084) | 0.009 |
| Age, years | 9 | 79.7 | (-0.503 to -0.059) | 0.020 |
| Female, % | 9 | 17.8 | (-0.234 to 0.059) | 0.203 |
| Glucocorticoids use, % | 7 | 37.1 | (-0.245 to 0.037) | 0.116 |
| Baseline lumbar spine BMD, g/cm^2^ | 7 | 4.4 | (-7.508 to 3.669) | 0.418 |
| Positive rheumatoid factor, % | 6 | 91.4 | (-0.286 to 0.035) | 0.097 |
| Denosumab dose | 8 studies with 12 arms | 27.7 | (-0.389 to 2.047) | 0.160 |
| BMD, bone mineral density; CI, Confidence interval; RA, rheumatoid arthritis | | | | |


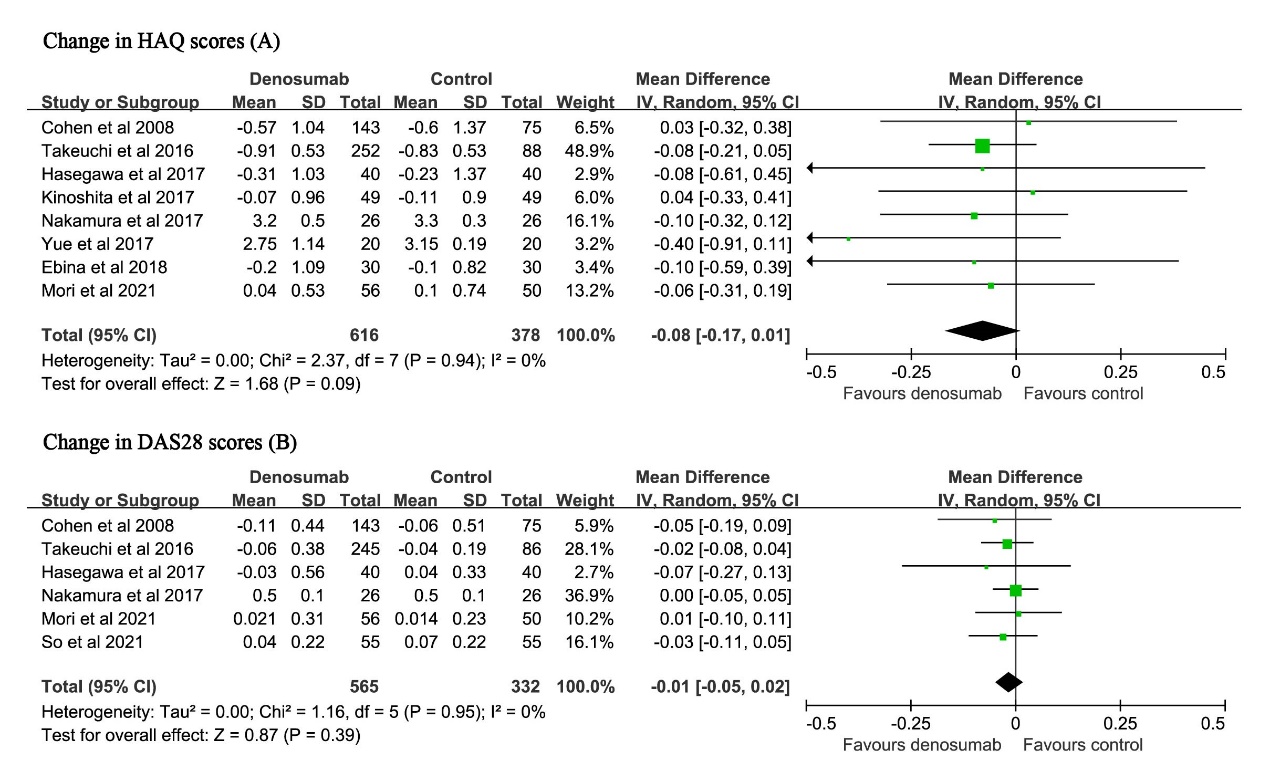


Figure S1. Forest plot for the effect of denosumab on the changes in HAQ scores (A) and DAS28 scores (B).


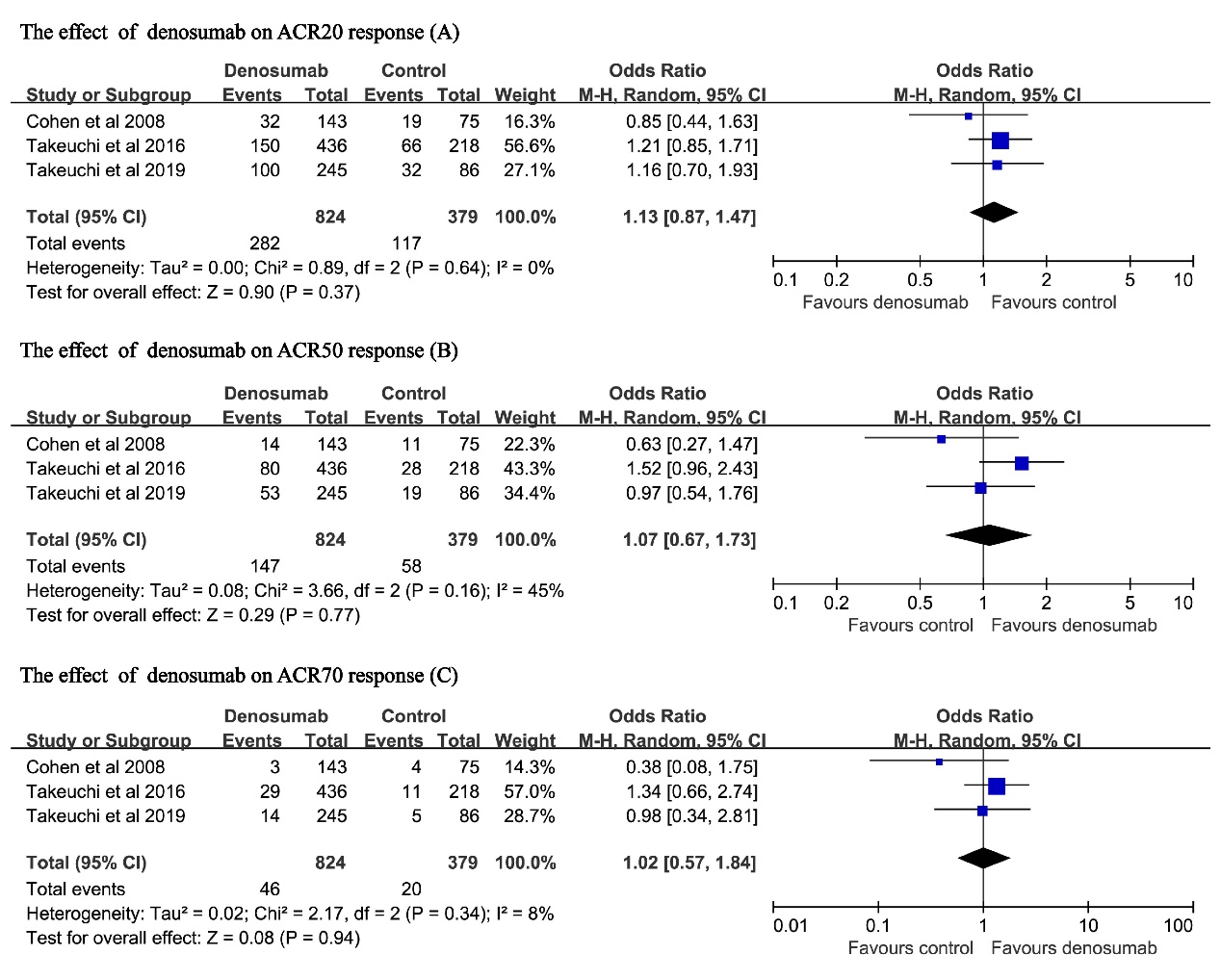


Figure S2. Forest plot for the effect of denosumab on ACR20 response (A), ACR50 response (B) and ACR70 response (C).


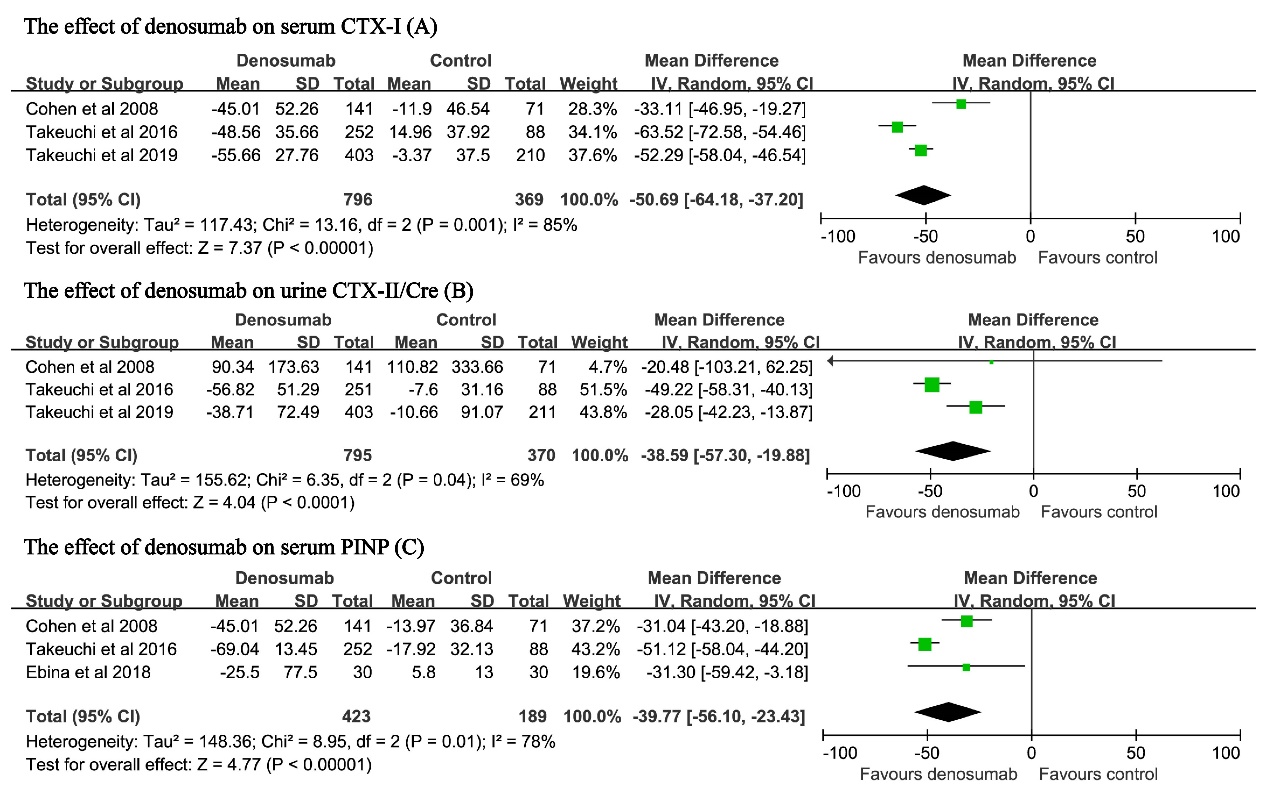


Figure S3. Forest plot for the effect of denosumab on serum CTX-I (A), urine CTX-II/creatinine (B) and serum PINP (C).


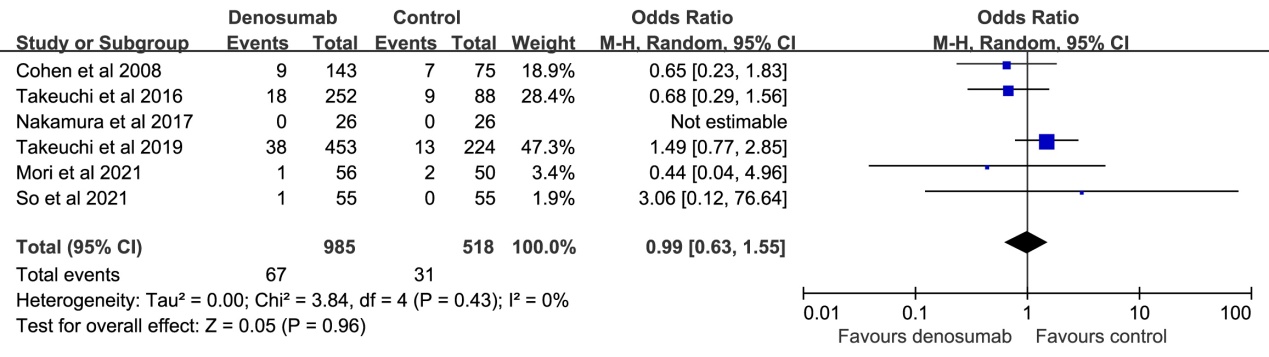


Figure S4. Forest plot for incidence rates of serious adverse events.


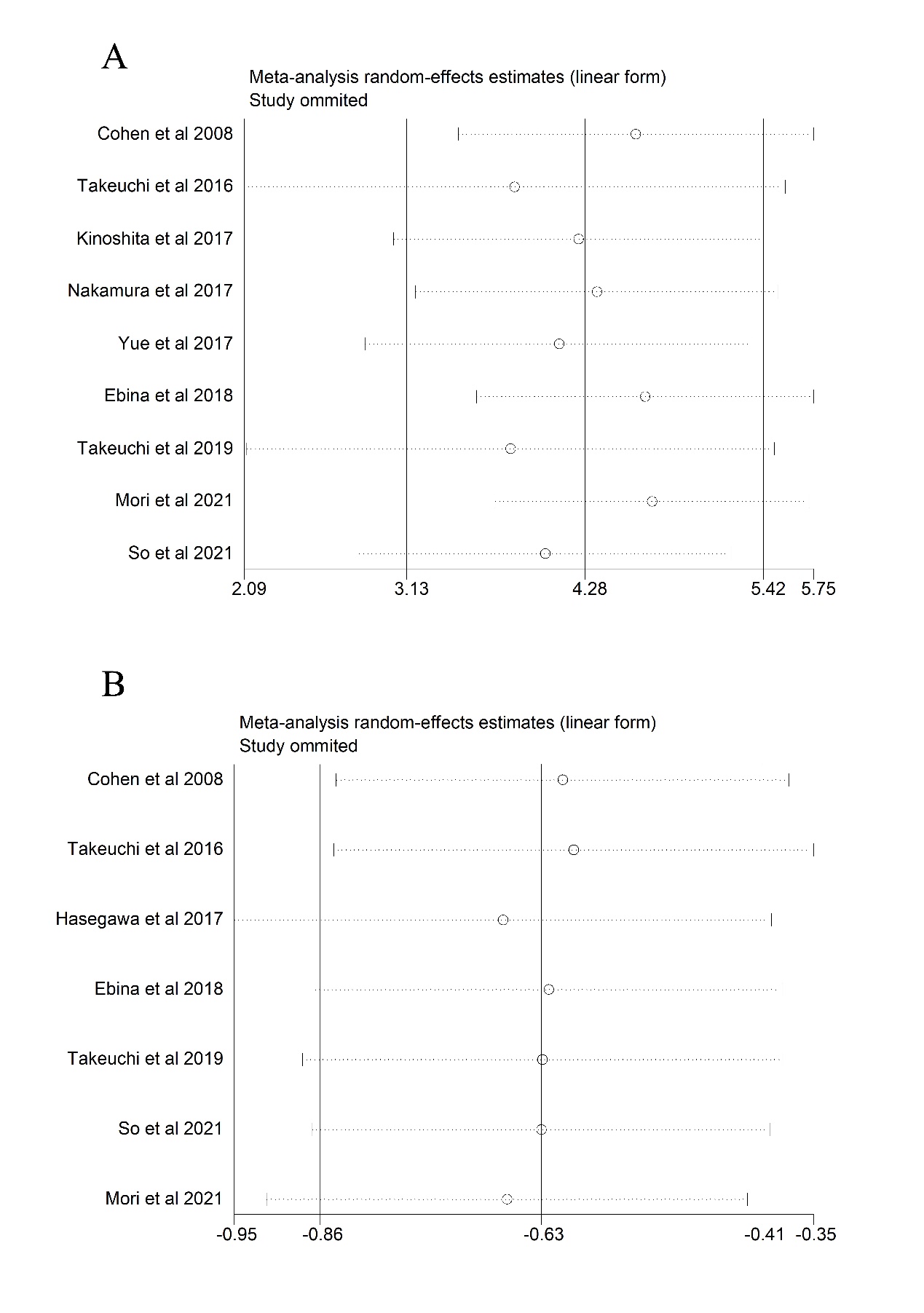


Figure S5. Sensitivity analysis for the effect of denosumab on the changes in lumbar spine BMD (A) and mTSS (B).


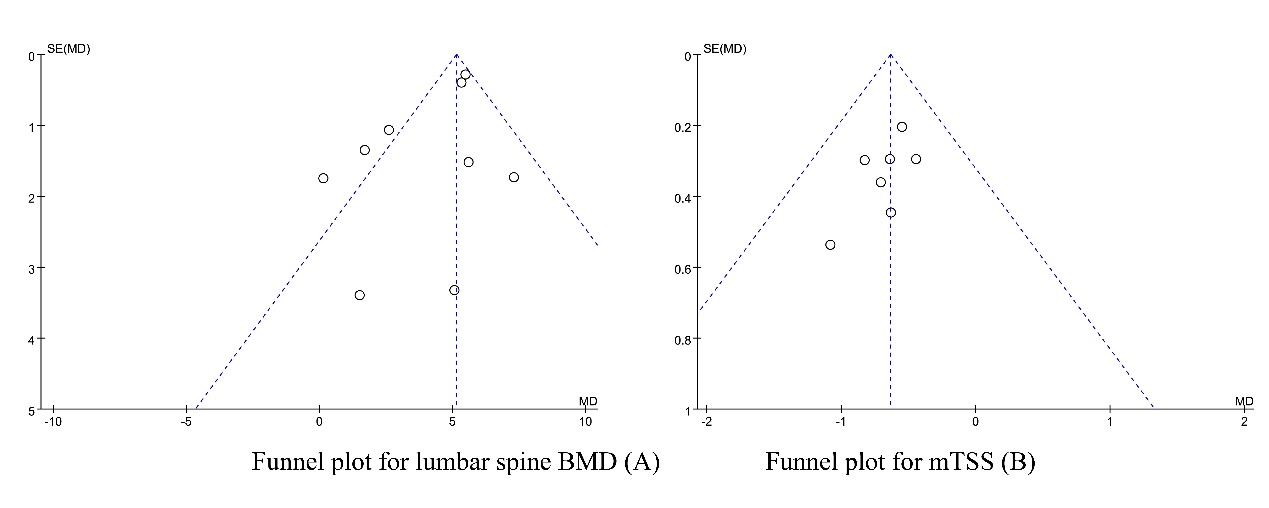


Figure S6. Funnel plots for the change in lumbar spine BMD (A) and mTSS (B).
